# Clinical characteristics and outcomes of critically ill coronavirus disease 2019 patients in Malaysia during the first wave: A multi-center observational study (COVIDICU-MY)

**DOI:** 10.3389/fmed.2022.1086288

**Published:** 2023-01-09

**Authors:** Noor Iftitah Ab Rahman, Nor’azim Mohd Yunos, Rafidah Atan, Jeevitha Mariapun, Mohd Ali‘Imran Ab Rahman, Abdul Jabbar Ismail, Shanti Rudra Deva

**Affiliations:** ^1^Department of Anaesthesiology, Faculty of Medicine, Universiti Malaya Medical Centre, Universiti Malaya, Kuala Lumpur, Malaysia; ^2^Clinical School Johor Bahru, Jeffrey Cheah School of Medicine and Health Sciences, Monash University Malaysia, Subang Jaya, Malaysia; ^3^Department of Social and Preventive Medicine, Faculty of Medicine, Universiti Malaya, Kuala Lumpur, Malaysia; ^4^Faculty of Medicine and Health Sciences, Universiti Malaysia Sabah, Kota Kinabalu, Sabah, Malaysia; ^5^Department of Anaesthesiology and Intensive Care, Hospital Kuala Lumpur, Kuala Lumpur, Malaysia

**Keywords:** COVID-19, critically ill, intubation, acute respiratory distress syndrome, mortality, prone

## Abstract

**Background:**

Coronavirus disease 2019 (COVID-19) emerged with a wide range of clinical presentations; Malaysia was not spared from its impact. This study describes the clinical characteristics of COVID-19 patients admitted to intensive care unit, their clinical course, management, and hospital outcomes.

**Methods:**

COVIDICU-MY is a retrospective analysis of COVID-19 patients from 19 intensive care units (ICU) across Malaysia from 1 March 2020 to 31 May 2020. We collected epidemiological history, demographics, clinical comorbidities, laboratory investigations, respiratory and hemodynamic values, management, length of stay and survival status. We compared these variables between survival and non-survival groups.

**Results:**

A total of 170 critically ill patients were included, with 77% above 50 years of age [median age 60, IQR (51–66)] and 75.3% male. Hypertension, diabetes mellitus, hyperlipidemia, chronic cardiac disease, and chronic kidney disease were most common among patients. A high Simplified Acute Physiology Score (SAPS) II score [median 45, IQR (34–49)] and Sequential Organ Failure Assessment (SOFA) score [median 8, IQR (6–11)] were associated with mortality. Patients were profoundly hypoxic with a median lowest PaO_2_/FiO_2_ ratio of 150 (IQR 99–220) at admission. 91 patients (53.5%) required intubation on their first day of admission, out of which 38 died (73.1% of the hospital non-survivors). Our sample had more patients with moderate Acute Respiratory Distress Syndrome (ARDS), 58 patients (43.9%), compared to severe ARDS, 33 patients (25%); with both ARDS classification groups contributing to 25 patients (54.4%) and 11 patients (23.9%) of the non-survival group, respectively. Cumulative fluid balance over 24 h was higher in the non-survival group with significant differences on Day 3 (1,953 vs. 622 ml, *p* < 0.05) and Day 7 of ICU (3,485 vs. 830 ml, *p* < 0.05). Patients with high serum creatinine, urea, lactate dehydrogenase, aspartate aminotransferase and d-dimer, and low lymphocyte count throughout the stay also had a higher risk of mortality. The hospital mortality rate was 30.6% in our sample.

**Conclusion:**

We report high mortality amongst critically ill patients in intensive care units in Malaysia, at 30.6%, during the March to May 2020 period. High admission SAPS II and SOFA, and severe hypoxemia and high cumulative fluid balance were associated with mortality. Higher creatinine, urea, lactate dehydrogenase, aspartate aminotransferase and d-dimer, and lymphopenia were observed in the non-survival group.

## Introduction

Coronavirus Disease 2019 (COVID-19), caused by the novel beta coronavirus SARS-CoV-2, has a wide range of clinical presentations–from being asymptomatic to having a mild illness, pneumonia, severe pneumonia, Acute Respiratory Distress Syndrome (ARDS), sepsis and septic shock (1). In February 2020, the World Health Organization (WHO) declared COVID-19 as a pandemic, and by 19 December 2021, the WHO Situation Report documented a COVID-19 global infection of more than 273 million people with a mortality of more than 5.3 million with Southeast Asia contributing to 44 million cases and more than 700 thousand deaths (2). On 25 December 2021, the official website of the Ministry of Health Malaysia reported a cumulative case of 2,741,179 COVID-19 positive patients with 2,778 daily cases and 306 active intensive care unit (ICU) cases (3). Understanding the more severely ill COVID-19 patients is necessary given the wide range of clinical presentations and the high mortality. A retrospective cohort data suggested that as high as 35% of patients could fall into the severe group and 28% critical (4).

Based on the published summary by the Chinese Center for Disease Control and Prevention, mortality risk factors identified included cardiovascular disease (10.5% of the death population), diabetes (7.3%), chronic respiratory disease (6.3%), hypertension (6.0%), and cancer (5.6%) (5). Among the highest risk groups in this report was the elderly population aged 70–79 years old, with a documented case fatality rate of 8%. The critically ill subgroup population had also been identified as having a higher risk of mortality; 49% in the same report mentioned above (5). A more focused retrospective cohort study done in two hospitals hardest hit with COVID-19 in Wuhan, China showed that older age, high Sequential Organ Failure Assessment (SOFA) score, and d-dimer greater than 1 μg/ml would identify patients with poor prognosis at an early stage (4). Another retrospective observational study in a specific population of 52 ICU patients in another hospital in Wuhan showed 61% mortality rate, with median days to death from ICU admission of 7 days and a higher risk of dying in the elderly (>65 years old) with comorbidities and those who fulfill criteria for ARDS (6).

In March 2020, there was a sudden surge of adult intensive care unit COVID-19 admissions in Malaysia (7). The unprecedented COVID-19 outbreak poses significant challenges to intensive care practice for a middle-income country with limited intensive care capacity and resources like Malaysia. This study aimed to describe the clinical characteristics of COVID-19 patients admitted to the Malaysian ICUs, their clinical course, management and hospital outcomes.

## Materials and methods

### Study design and population

We conducted a retrospective analysis of data from 19 participating ICUs across Malaysia for a period of 2 months from 1 March 2020 to 31 May 2020. We included patients with confirmed positive COVID-19 reverse transcription polymerase chain reaction (RT-PCR) tests either before or after their ICU admissions.

### Variables and outcomes

We collected data of socio-demography, admission characteristics including the severity scores, laboratory and radiographic investigations, management and clinical outcomes. The outcomes measured were duration of mechanical ventilation, tracheotomy, ICU length of stay and hospital length of stay. These variable and outcomes were then compared between the non-survived and survived groups.

### Data extraction

We extracted data from paper and electronic resources available and documented in pre-designed case report forms (CRFs). The data for CRFs of each participating centre were collected by assigned clinicians who were experienced with intensive care practices.

There were three CRFs: the core CRF, the daily CRF, and the summary CRF. The core CRF consisted of baseline information on hospital and ICU admission i.e., epidemiological history, demographics, vital signs, comorbidities, admission date, hospital admission signs and symptoms, confirmatory pathogen testing, laboratory investigations and severity scores based on admission SAPS II and SOFA.

The daily CRF consisted of clinical observation data i.e., types of respiratory support, PaO_2_ values, need for prone positioning, worst hemodynamic values, and highest vasopressor dose. We also included data of fluids input, output and balance, renal replacement therapy and laboratory values.

The summary CRF included the discharge details–dead or alive status, length of ICU stay, oxygen and renal support required upon discharge, tracheostomy and length of hospital stay.

### Ethics

We received ethics approval from the Medical Research Ethics Committee of Malaysian Ministry of Health (NMRR-20-1037-54772-IIR) and University of Malaya Medical Centre (202049-8484). Informed consent was waived as the study was observational and the data were de-identified.

### Statistical analysis

Descriptive data are expressed as the median and interquartile range (IQR) for continuous data and numbers and percentages for categorical data. Associations between survival groups were examined using the Pearson’s chi-squared test or Fisher’s exact test for categorical data and the Wilcoxon rank-sum test for continuous data. A value of *P* < 0.05 is considered statistically significant. All analysis were performed with Stata version 17. Missing or unknown data were excluded from the analysis.

## Results

### Demographics and clinical characteristics

A total of 170 patients from 19 hospitals in Malaysia were included during the study period for analysis. A total of 52 patients died during hospitalization, and 118 were discharged. Majority of the patients were above 50 years old (77%) with a median age of 60 (IQR 51–66), and most patients were male (75.3%) (Table 1). A total of 137 (80.6%) patients were of Malay ethnicity, which represents the most of Malaysia’s population by ethnicity (8). This was followed by patients of Chinese ethnicity, 11.8%, and Indian, 3.5%, other Bumiputera, 1.2%, and foreigners, 2.9%. Most patients had close contact with a confirmed COVID-19 case (40%), a history of presence in large social gatherings (39.8%), and a history of traveling to an area with documented confirmed COVID-19 cases (34.7%). It is worth noting that there were cases with a history of presence in a healthcare facility (17.5%) and a history of laboratory handling of confirmed COVID-19 samples (11%).

**TABLE 1:** Demographics, epidemiological factors, comorbidities, clinical investigations, and symptoms within 24 h at presentation/Admission at the hospital of coronavirus disease 2019 (COVID-19) intensive care unit patients.

Characteristics	*n* ^c^	All patients	Non-survival	Survival	*P*-value
Age (years)^a^	170	60 (51–66)	62.0 (51.5–68.5)	60 (51–65)	0.138
Age categories (years)	170	–	–	–	0.403
≤30	–	3 (1.8)	0 (0.0)	3 (2.5)	–
31–50	–	36 (21.2)	10 (19.2)	26 (22.0)	–
51–70	–	108 (63.5)	32 (61.5)	76 (64.4)	–
>70	–	23 (13.5)	10 (19.2)	13 (11.0)	–
Sex	170	–	–	–	0.953
Male	–	128 (75.3)	39 (75.0)	89 (75.4)	–
Female	–	42 (24.7)	13 (25.0)	29 (24.6)	–
Ethnicity	170	–	–	–	0.226
Malay	–	137 (80.6)	41 (78.9)	96 (81.4)	–
Chinese	–	20 (11.8)	9 (17.3)	11 (9.3)	–
Indian	–	6 (3.5)	0 (0.0)	6 (5.1)	–
Other Bumiputera	–	2 (1.2)	0 (0.0)	2 (1.7)	–
Foreigner	–	5 (2.9)	2 (3.9)	3 (2.5)	–
A history of presence in large social gathering^b^	118	47 (39.8)	14 (42.4)	33 (38.8)	0.720
A history of travel to an area with documented cases of COVID-19 infection^b^	124	43 (34.7)	11 (31.4)	32 (36.0)	0.634
Close contact with a confirmed case of COVID-19 infection, while that patient was asymptomatic^b^	115	46 (40.0)	16 (47.1)	30 (37.0)	0.317
Presence in healthcare facility where COVID-19 infections have been managed^b^	120	21 (17.5)	9 (25.7)	12 (14.1)	0.129
Presence in a laboratory handling of confirmed COVID-19 samples^b^	118	13 (11.0)	5 (14.7)	8 (9.5)	0.517
Co-morbidities^b^	–	–	–	–	–
Chronic cardiac disease	134	20 (14.9)	10 (23.8)	10 (10.9)	0.051
Hypertension	136	79 (58.1)	27 (64.3)	52 (55.3)	0.328
Chronic pulmonary disease	136	1 (0.7)	0 (0.0)	1 (1.1)	1.000
Asthma	136	4 (4.3)	0 (0.0)	4 (4.3)	0.311
Chronic kidney disease	134	20 (14.9)	9 (22.5)	11 (11.7)	0.108
Moderate/severe liver disease	136	0 (0.0)	0 (0.0)	0 (0.0)	–
Mild liver disease	135	0 (0.0)	0 (0.0)	0 (0.0)	–
Chronic neurological disorder	136	0 (0.0)	0 (0.0)	0 (0.0)	–
Malignant neoplasm	135	2 (1.5)	2 (4.8)	0 (0.0)	0.095
Chronic hematologic disease	136	0 (0.0)	0 (0.0)	0 (0.0)	–
AIDS/HIV	131	2 (1.5)	0 (0.0)	2 (2.2)	1.000
Obesity	134	17 (12.7)	6 (14.6)	11 (11.8)	0.653
Diabetes	135	60 (44.4)	19 (46.3)	41 (43.6)	0.770
Rheumatologic disorder	136	5 (3.7)	4 (9.5)	1 (1.1)	0.032*
Dementia	136	0 (0.0)	0 (0.0)	0 (0.0)	–
Malnutrition	135	0 (0.0)	0 (0.0)	0 (0.0)	–
Hyperlipidemia	130	31 (23.9)	12 (30.8)	19 (20.9)	0.225
Smoking^b^	124	4 (3.2)	3 (8.3)	1 (1.1)	0.073
Pregnant^b^	139	1 (0.7)	0 (0.0)	1 (0.9)	1.000
BMI (kg/m^2^)^a^	70	26.7 (24.8–30.5)	27.7 (24.2–30.5)	26.0 (25.0–31.1)	0.945
Systolic BP (mmHg)^a^	136	134.0 (121.0–149.0)	132.5 (119.0–151.0)	134.5 (122.0–148.0)	0.699
Diastolic BP (mmHg)	136	75 (65–84)	72 (61–81)	77 (67–85)	0.044*
Pulse rate (beats/min)^a^	136	92.0 (82.5–105.0)	96 (82–118)	90.5 (83.0–99.0)	0.062
Pulse ł 125 beats/min	136	6 (4.4)	4 (9.5)	2 (2.1)	0.073
Capillary refill time (>2 s)^b^	101	3 (3.0)	1 (2.0)	2 (1.9)	0.115
Respiratory rate (breaths/min)^a^	128	22 (20–28)	23 (19–30)	22 (20–28)	0.756
Respiratory rate > 24 breaths/min	128	51 (39.8)	19 (45.2)	32 (37.2)	0.384
Oxygen saturation (%)^a^	126	97 (95–99)	97.0 (92.5–99.0)	97 (95–99)	0.685
Oxygen requirement^b^	137	96 (70.1)	30 (71.4)	66 (69.5)	0.818
Temperature (^°^C)^a^	133	37.4 (36.9–38.0)	37.0 (36.7–38.0)	37.5 (37.0–38.0)	0.329
Clinical investigations^a^	–	–	–	–	–
Hemoglobin (g/L)	135	13.3 (11.6–14.9)	13.0 (10.9–13.9)	13.7 (12.0–15.0)	0.068
White blood cell count, × 10^9^/L	135	7.0 (5.6–9.6)	7.6 (5.6–9.6)	6.9 (5.8–9.5)	0.886
Lymphocyte count, (cells/μl)	119	1.2 (0.9–1.6)	1.0 (0.9–1.4)	1.2 (0.9–1.7)	0.125
Neutrophil (cells/μl)	111	5.5 (3.9–9.8)	6.0 (3.9–9.8)	5.3 (4.0–9.7)	0.911
Hematocrit (%)	115	39.9 (34.7–44.1)	38.4 (31.1–43.5)	40.3 (35.1–44.4)	0.178
Platelet count, × 10^9^/L	132	222 (169.5–288)	222 (163.5–292.5)	220.5 (171.5–281.0)	0.949
Total bilirubin, μmol/L	170	8.5 (1.0–28.0)	9.5 (1.0–27.0)	8.0 (1.0–28.0)	0.676
Serum creatinine, μmol/L	133	93 (77–147)	111.5 (78.5–240.0)	90 (77–121)	0.021*
Serum urea, mmol/L	134	5.7 (4.1–13.4)	7.2 (4.7–18.7)	5.2 (3.9–8.2)	0.004*
LDH, UL	98	424 (297.5–602.5)	599.9 (378.5–719.4)	346 (378.5–719.4)	0.002*
AST, UL	117	48 (30.5–74)	57 (43–109)	45 (29–69.75)	0.028*
Chest radiography	113	–	34	79	0.130
Interstitial opacities	–	30 (26.4)	12 (35.3)	18 (22.8)	–
Ground glass opacities	–	45 (39.9)	8 (23.5)	37 (46.8)	–
Consolidation	–	36 (31.9)	13 (38.2)	23 (29.1)	–
Nodular opacities	–	2 (1.8)	1 (2.9)	1 (1.3)	–
Severity score on ICU admission^a^	–	–	–	–	–
SAPS II	95	29 (18–45)	45 (34–49)	21.5 (15.0–31.0)	<0.001*
SOFA	95	5 (2–8)	8 (6–11)	3 (2–6)	<0.001*

Values are number (percentage) unless indicated otherwise; Percentages may not total to 100% due to rounding.

^a^Values are median (IQR).

^b^Percentage was calculated from a total based on the “Yes” and “No” categories (missing or unknown data were excluded).

^c^The total number of patients with data available for each variable. *P*-values for continuous variables were obtained using the Wilcoxon rank-sum test. *P*-values for categorical variables were obtained using the Pearson’s chi-squared test or Fisher’s exact test.

*Statistically significant (*p* < 0.05).

BMI, body mass index; BP, blood pressure; SAPS II score, simplified acute physiology score; SOFA score, sequential organ failure assessment score.

There were many comorbidities reported among our patients with the most common being hypertension (58.1%) and diabetes (44.1%). At hospital presentation, the median Body Mass Index (BMI) of all patients was 26.7 (IQR 24.8–30.5), median systolic blood pressure 134 mmHg (IQR 121–149), diastolic 75 mmHg (IQR 65–84), pulse rate 92 beats/min (IQR 82.5–105), respiratory rate 22 breaths/min (IQR 20–28) and temperature 37.4 degrees of Celsius (IQR 36.9–38.0). 70% of all patients received oxygen supplementation at admission, with the median oxygen saturation 97% (IQR 95–99). There was no difference between survivors and non-survivors for these co-morbidities and clinical parameters.

Several blood investigations showed clinically different levels in the non-survivors compared to survivors at hospital or ICU admissions. These were creatinine (111.5 vs. 90 μmol/L, *P* = 0.021) and lactate dehydrogenase (599.9 vs. 346 UL, *P* = 0.002).

The median SAPS II score on ICU admission for all patients was 29, and those that died had a significantly higher median SAPS II of 45 compared to the survival group’s score of 21.5 (*P* < 0.001). The median SOFA score for all patients was five and those that died had a significantly higher median SOFA score of eight compared to a SOFA score median of three for the survival group (*P* < 0.001).

### Respiratory characteristics and support during first 24 h of ICU admission

In the first 24 h of ICU admission, 17 (10%) patients received oxygen *via* nasal prong while 49 patients received oxygen *via* different types of facemasks; 15 (30.6%) *via* Hudson facemask, 26 (53.1%) *via* non-rebreathing mask and 8 (16.3%) *via* Venturi mask (Table 2). A total of 91 patients had to be intubated in the first 24 h of ICU with a clinically significant number of them died while in the hospital, 38 (73.1% of the non-survival group) compared to 53 patients who survived (44.9% of the survival group); *P* = 0.256. The most common ventilation modes used for all patients were Synchronised Intermittent Mandatory Ventilation (SIMV) (63.5%) and Assist Control (AC) (16.5%). The median positive end-expiratory pressure, PEEP, that was used was 12 cmH_2_O (IQR 10–12.5).

**TABLE 2:** Clinical and respiratory support characteristics during the first 24 h in the intensive care unit (ICU).

Characteristics	*n* ^c^	All patients	Non-survival	Survival	*P*-value
Nasal prong, (3 L/min)	170	17 (10.0)	2 (3.9)	15 (12.7)	0.076
Type O_2_ mask	49	–	–	–	0.278
Face mask/hudson mask	–	15 (30.6)	1 (10.0)	14 (35.9)	–
High-flow non-rebreathing face mask	–	26 (53.1)	7 (70.0)	19 (48.7)	–
Venturi mask	–	8 (16.3)	2 (20.0)	6 (15.4)	–
High-flow nasal cannula^a^	7	1 (1–1), *n* = 7	1 (1–1), *n* = 1	1 (1–1), *n* = 6	1.000
Mode of ventilation	91	–	38	53	0.433
Synchronized intermittent mandatory ventilation (SIMV)	–	60 (65.9)	28 (73.7)	32 (60.4)	–
Airway pressure release ventilation (APRV)	–	5 (5.5)	3 (7.9)	2 (3.8)	–
Assist-control (AC)	–	15 (16.5)	5 (13.2)	10 (18.9)	–
Bilevel ventilation	–	5 (5.5)	1 (2.6)	4 (7.6)	–
Pressure support ventilation (PSV)/continuous positive airway pressure (CPAP)	–	6 (6.6)	1 (2.6)	5 (9.4)	–
Positive end-expiratory pressure (PEEP), cmH_2_O	84	12.0 (10.0–12.5)	12.0 (11.0–14.0), *n* = 35	11.0 (10.0–12.0), *n* = 49	0.010*
Neuromuscular blocking agents	94	18 (19.2)	9 (25.0)	9 (15.5)	0.256
Intubated	170	91 (53.5)	38 (73.1)	53 (44.9)	0.001*
Acute respiratory distress syndrome (ARDS), mm Hg	132	–	–	–	0.146
≤300 (none)	–	16 (12.1)	2 (4.4)	14 (16.3)	–
≥200 to <300 (mild)	–	25 (18.9)	8 (17.4)	17 (19.8)	–
≤100 to <200 (moderate)	–	58 (43.9)	25 (54.4)	33 (38.4)	–
<100 (severe)	–	33 (25.0)	11 (23.9)	22 (25.6)	–
Lowest PF (PaO_2_/FiO_2_) ratio^a^	132	150.4 (99.9–220.0)	128.3 (100.4–187.8), *n* = 46	166.8 (99.3–250.0), *n* = 86	0.030*
Prone positioning	161	26 (16.2)	4 (8.2)	22 (19.6)	0.069
Renal replacement therapy (RRT)^b^	159	11 (6.9)	5 (10.2)	6 (5.5)	0.276

Values are number (percentage) unless indicated otherwise; Percentages may not total to 100% due to rounding.

^a^Values are median (IQR).

^b^Percentage was calculated from a total based on the “Yes” and “No” categories (missing or unknown data were excluded).

^c^The total number of patients with data available for each variable. PaO_2_, arterial partial pressure of oxygen (mmHg); FiO_2_, the fraction of inspired oxygen. The acute respiratory distress syndrome (ARDS) categories are based on the values of the lowest PF ratio (PaO_2_/FiO_2_). *P*-values for continuous variables were obtained using the Wilcoxon rank-sum test. P-values for categorical variables were obtained using the Pearson’s chi-squared test or Fisher’s exact test.

*Statistically significant (*p* < 0.05).

Out of 132 patients analyzed for ARDS, 25 had mild ARDS (18.9%), 58 had moderate ARDS (43.9%), and 33 had severe ARDS (25%). 25 patients in the moderate ARDS group and 11 patients in the severe ARDS group died, contributing to 54.4 and 23.9% of the non-survival group. The lowest PaO_2_/FiO_2_ (PF) ratio median value was 150 (IQR 99.6–220), with a significant difference between the non-survival group (median value 128.3) and the survival group (median value 166.8), *P* = 0.030.

### Lowest PaO_2_/FiO_2_ ratio, cumulative fluid balance and maximum norepinephrine dose for days 1, 3, and 7 of ICU

We analysed these clinical variables on days 1, 3, and 7 in the ICU, as shown in Table 3. The significant difference in median PF ratio between the non-survival group and survival group seen in the first 24 h (Day 1 of ICU) above was also seen on Day 3.

**TABLE 3:** Physiological measurements for days 1, 3, and 7 in the intensive care unit (ICU).

	*n* ^a^	All patients	Non-survival	Survival	*P*-value
Lowest PaO_2_/FiO_2_ ratio (mmHg)	–	–	–	–	–
Day 1	132	150.4 (99.9–220.0)	128.3 (100.4–187.8)	166.8 (99.3–250.0)	0.031*
Day 3	118	164.3 (120.0–217.5)	136.3 (98.4–204.0)	179.0 (134.8–220.0)	0.016*
Day 7	75	181.5 (137.8–234.7)	178.1 (109.8–235.6)	181.5 (142.0–234.7)	0.710
Cumulative fluid balance in 24 h (ml)	–	–	–	–	–
Day 1	162	336.5 (4.0–741.0)	505.5 (34.0–890.3)	258.0 (4.0–705.0)	0.185
Day 3	153	1097.0 (230.0–2431.0)	1953.0 (946.0–2252.5)	622.0 (-7.0 to 1855.0)	<0.001*
Day 7	134	2141.0 (188.0–3953.0)	3485.3 (2141.0–5660.1)	830.5 (-43.4 to 3077)	<0.001*
Maximum norepinephrine dose in 24 h (mcg/kg/min)	–	–	–	–	–
Day 1	55	0.19 (0.10–0.53)	0.26 (0.16–0.65)	0.10 (0.10–0.30)	0.030*
Day 3	53	0.10 (0.07–0.30)	0.17 (0.07–0.50)	0.10 (0.07–0.20)	0.817
Day 7	28	0.08 (0.05–0.50)	0.08 (0.08–0.50)	0.05 (0.05–0.50)	0.266

Values are median (IQR).

^a^The total number of patients with data available.

PaO_2_, arterial partial pressure of oxygen (mmHg); FiO_2_, the fraction of inspired oxygen. *P*-values were obtained using the Wilcoxon rank-sum test.

*Statistically significant (*p* < 0.05).

There was significantly higher cumulative fluid balance among the non-survival group compared with the survival group on Day 3 (1,953 ml vs. 622 ml, *P* < 0.001) and on Day 7 (3,485 ml vs. 830 ml, *P* < 0.001). With regard to norepinephrine dose, there was a significant difference seen on Day 1 of ICU (non-survival 0.26 mcg/kg/min vs. survival 0.1 mcg.kg.min, *P* 0.03).

### PaO_2_/FiO_2_ ratio for patients on prone position from day 1 to day 7

For a small number of patients put on prone positioning and had adequate PaO_2_ and FiO_2_ data, we compiled the changes in the PF ratio before and after the prone (Table 4). There was a significant difference before and after the prone position from Day 1 to Day 3. The PF ratios before prone were 88, 95, and 138 mmHg for Days 1, 2, and 3, respectively. After prone, the PF ratios for Days 1, 2, and 3 were significantly higher 173.2, 242, and 216 mmHg, respectively. From Day 4 to Day 7, the difference between before and after prone was no longer significant and there were also fewer patients with adequate PaO_2_ and FiO_2_ data for comparison.

**TABLE 4:** PaO_2_/FiO_2_ ratio (mmHg) for coronavirus disease 2019 (COVID-19) intensive care unit patients on prone position in the intensive care unit (ICU) from days 1 to 7.

Day	*n* ^a^	Before prone position (PaO_2_/FiO_2_)	During prone position (PaO_2_/FiO_2_)	P/F ratio difference	*P*-value^b^
1	15	88.0 (79.0–163.4)	173.2 (133.8–305.0)	71.0 (-14.0 to 170.0)	0.009*
2	11	95.0 (83.0–254.0)	242.0 (236.0–281.7)	109.8 (-14.7 to 191.7)	0.026*
3	15	138.0 (121.0–187.1)	216.0 (172.8–284.0)	46.8 (16.0–82.9)	0.001*
4	7	150.0 (93.8–190.5)	212.6 (141.0–250.0)	34.2 (-70.4 to 100.4)	0.612
5	6	171.2 (121.0–292.0)	220.8 (150.0–291.4)	23.5 (-62.8 to 74.3)	0.753
6	3	140.5 (86.1–215.7)	183.1 (180.0–220.7)	42.6 (5.0–93.9)	0.109
7	6	124.8 (96.8–238.0)	152.0 (130.5–272.3)	33.0 (27.8–72.3)	0.173

Values are median (IQR).

^a^The total number of patients with data available.

PaO_2_, arterial partial pressure of oxygen (mmHg); FiO_2_, the fraction of inspired oxygen;. P/F ratio difference, P/F ratio of during prone position–P/F ratio of before prone position.

^b^*P*-values for the P/F ratio difference were obtained using the Wilcoxon signed-rank test.

*Statistically significant (*p* < 0.05).

### Clinical outcomes

There was no difference in the duration of mechanical ventilation between the survival group (median 6.8 days, IQR 2.7–15.5) and the non-survival group (median 5.4 days, IQR 2.7–15.5); *P* = 0.869 (Table 5). There was also no difference in the length of ICU stay; the survival group had a median ICU stay of 7.5 days (IQR 3.5–15.5) and the non-survival group 6.5 days (IQR 3.0–15.0); *P* = 0.571. However, there was a significant difference in the hospital length of stay between the groups, survival group had a median of hospital stay 19 days (IQR 13–32.5) and non-survival group 13 days (IQR 7–24), *P* = 0.001.

**TABLE 5:** Clinical outcomes of coronavirus disease 2019 (COVID-19) intensive care unit patients.

Characteristics	*n* ^c^	All patients	Non-survival	Survival	*P*-value
Duration of mechanical ventilation, days^a^	59	6.6 (2.5–12.5)	5.4 (2.7–15.5), *n* = 15	6.8 (2.3–12.0), *n* = 44	0.869
Tracheotomy^b^	96	2 (2.1)	1 (3.3)	1 (1.5)	0.530
ICU length of stay, days^a^	168	7.3 (3.0–15.0)	7.5 (3.5–15.5), *n* = 51	6.5 (3.0–15.0), *n* = 117	0.571
Hospital length of stay, days^a^	157	17 (11–28)	13 (7–24), *n* = 49	19 (13–32.5), *n* = 108	0.001*

^a^Values are median (IQR).

^b^Values are numbers (%).

^c^The total number of patients with data available.

*Statistically significant (*p* < 0.05).

### Supplementary results

Lymphocyte counts were generally low for both groups at admission and generally improved over time for survivors, but for non-survivors continued to dip and, toward the end, soared to a much higher level (Supplementary Figure 1). As per Table 1, we found that there were significant differences in some markers, and we tracked them over 14 days; ferritin, lactate dehydrogenase (LDH), d-dimer, creatinine, urea, and amino aspartate (AST) were generally higher in non-survival throughout 14 days in ICU. However, we only found significant differences between the groups for up to Day 2 for procalcitonin and AST, up to Day 4 for 165 d-dimer and LDH, and as long as Day 12 for urea and creatinine.

## Discussion

### Key findings

We analyzed data of 170 critically ill patients with confirmed COVID-19 infection admitted to ICUs during the first wave of COVID-19 outbreak between March and May 2020. A total of 52 (30.6%) of the critically ill patients died, and 118 (69%) were discharged.

Our analysis reveals that the non-survival group had higher SAPS II and SOFA scores on ICU admission, lower PF ratio on Days 1 and 3 of ICU, and higher cumulative balance on Days 3 and 7 of ICU. Patients in the non-survival group were also observed to have significantly higher ferritin, lactate dehydrogenase (LDH), d-dimer, creatinine, urea, and amino aspartate (AST) levels throughout their ICU stay.

### Strengths of the study

This was the first and only study on a multi-centre COVID-19 ICU patients in Malaysia. Its main strength lies on the valuable record of clinical characteristics of critically ill COVID-19 patients in a low–and middle-income (LMIC) country, and how the intensive care practice in such setting managed the ever-challenging early wave of COVID-19.

### Limitations of the study

The biggest limitation of this study is its incomplete data acquisition. There were many constraints that led to this. These include the lack of electronic medical record facilities in most hospitals and the overwhelming burden of COVID-19 to the nationwide ICU healthcare teams during the period of March to May 2020. We also acknowledge that while we collected data from multiple ICUs across the country, the small sample in our study may limit the generalizability of the results.

### Comparison with previous studies

Our sample’s ICU mortality rate of 30.6% reflected the high risk of mortality in the critically ill patients in Malaysia, with a study on all adult COVID-19 hospital admissions in 18 designated centres at about the same period only showing a case fatality rate of 1.2% (8). Comparatively, in the European countries, a study by the REVA network group reported 31% mortality (9) among the critically ill while a study in Wuhan reported 61.5% mortality (6). On another note, our finding of a median SOFA score of eight on Day 1 of ICU in the non-survival group was comparatively higher than other reported mortality-associated SOFA scores, namely a median of 4.5 that was reported by Wang et al. (10) in Wuhan and a median score of six by Yang et al. (6).

Our 53.5% rate of intubation and mechanical ventilation on the first day of ICU admission was comparable to the study in Wuhan by Wang et al. (10) at 47% and Yang et al. (6) at 42%. The REVA network group reported that 63% of patients required intubation in their first 24 h of ICU admission, and 80% required intubation during their ICU stay (9). On the other hand, in Lombardy, Italy, the rate of ICU intubation was 88% (11).

Our hypoxemia findings in the non-survival group were consistent with observations made in other COVID-19 ICU cohorts (12). With regard to ARDS, our findings that 54.4% of the non-survivors had moderate ARDS and 23.9% severe ARDS reflect a higher mortality rate for ARDS compared to Bellani et al. (13) in the LUNG SAFE study, in which the mortality rate was close to 50%.

Our biochemistry findings, on the other hand, are also consistent with the observations by Zhou et al. (4) and Yang et al. (6) in Wuhan, who reported lymphopenia coupled with raised inflammatory markers, increased liver transaminases, and serum creatinine in the severely diseased group.

### Significance of study findings

Our findings demonstrate comparable clinical characteristics and outcomes in our low–and middle-income country setting. The similar patterns of ICU severity scores, respiratory and biochemistry presentations, and the clinical outcomes seen in our population importantly add to the body of knowledge surrounding the global impact and response to the COVID-19 pandemic.

## Conclusion

In conclusion, we reported high mortality among critically ill patients in multi-intensive care units in Malaysia during the first wave of COVID-19 between March and May 2020. Most patients were above 50 years old with a prevalence of comorbidities. Higher SAPS II and SOFA scores on ICU admission, severe hypoxemia and higher cumulative fluid balance were associated with mortality in our sample. We also found the non-survivors to have significantly higher ferritin, lactate dehydrogenase (LDH), d-dimer, creatinine, urea, and amino aspartate (AST) levels throughout their ICU stay.

## Data availability statement

The raw data supporting the conclusions of this article will be made available by the authors, without undue reservation.

## Ethics statement

This study was approved by the Medical Research Ethics Committee of the Malaysian Ministry of Health (NMRR-20-1037-54772-IIR) and the University of Malaya Medical Centre (202049-8484). Informed consent was waived as the study was observational and the data were deidentified.

## Author contributions

NY: full access to all the data in the study, takes responsibility for the integrity of the data, the accuracy of the data analysis, and critical revision of the manuscript for important intellectual content. NY and RA: study concept and design and acquisition of data. JM, NA, MA, and RA: analysis and interpretation of data. NA and JM: drafting of the manuscript. JM: statistical analysis. SD and NY: obtained funding. SD and AI: administrative, technical, or material support. All authors contributed to the article and approved the submitted version.
